# A urine-based DNA methylation assay to facilitate early detection and risk stratification of bladder cancer

**DOI:** 10.1186/s13148-021-01073-x

**Published:** 2021-04-26

**Authors:** Weimei Ruan, Xu Chen, Ming Huang, Hong Wang, Jiaxin Chen, Zhixin Liang, Jingtong Zhang, Yanqi Yu, Shang Chen, Shizhong Xu, Tianliang Hu, Xia Li, Yuanjie Guo, Zeyu Jiang, Zhiwei Chen, Jian Huang, Tianxin Lin, Jian-Bing Fan

**Affiliations:** 1 AnchorDx Medical Co., Ltd, Unit 502, 3rd Luoxuan Road, International Bio-Island, Guangzhou, 510300 China; 2 Department of Urology, Sun Yat-Sen Memorial Hospital, Sun Yat-Sen University, Guangzhou, 510120 China; 3 Department of Pathology, School of Basic Medical Science, Southern Medical University, Guangzhou, 510515 China; 4 AnchorDx, Inc., 46305 Landing Pkwy, Fremont, CA 94538 USA; 5 Guangdong Provincial Key Laboratory of Malignant Tumor Epigenetics and Gene Regulation, Sun Yat-Sen Memorial Hospital, Sun Yat-Sen University, Guangzhou, 510120 China

**Keywords:** Bladder cancer, DNA methylation assay, Early detection, Risk stratification

## Abstract

**Background:**

Current non-invasive tests have limited sensitivities and lack capabilities of pre-operative risk stratification for bladder cancer (BC) diagnosis. We aimed to develop and validate a urine-based DNA methylation assay as a clinically feasible test for improving BC detection and enabling pre-operative risk stratifications.

**Methods:**

A urine-based DNA methylation assay was developed and validated by retrospective single-center studies in patients of suspected BC in Cohort 1 (*n* = 192) and Cohort 2 (*n* = 98), respectively. In addition, a prospective single-center study in hematuria patient group (Cohort 3, *n* = 174) was used as a second validation of the model.

**Results:**

The assay with a dual-marker detection model showed 88.1% and 91.2% sensitivities, 89.7% and 85.7% specificities in validation Cohort 2 (patients of suspected BC) and Cohort 3 (patients of hematuria), respectively. Furthermore, this assay showed improved sensitivities over cytology and FISH on detecting low-grade tumor (66.7–77.8% vs. 0.0–22.2%, 0.0–22.2%), Ta tumor (83.3% vs. 22.2–41.2%, 44.4–52.9%) and non-muscle invasive BC (NMIBC) (80.0–89.7% vs. 51.5–52.0%, 59.4–72.0%) in both cohorts. The assay also had higher accuracies (88.9–95.8%) in diagnosing cases with concurrent genitourinary disorders as compared to cytology (55.6–70.8%) and FISH (72.2–77.8%). Meanwhile, the assay with a five-marker stratification model identified high-risk NMIBC and muscle invasive BC with 90.5% sensitivity and 86.8% specificity in Cohort 2.

**Conclusions:**

The urine-based DNA methylation assay represents a highly sensitive and specific approach for BC early-stage detection and risk stratification. It has a potential to be used as a routine test to improve diagnosis and prognosis of BC in clinic.

**Supplementary Information:**

The online version contains supplementary material available at 10.1186/s13148-021-01073-x.

## Background

Bladder cancer (BC) is the most common malignancy of the urinary tract and one of the leading causes of cancer death in men [1]. Based on the aggressiveness and treatment modalities, BC can be categorized as muscle invasive BC (MIBC) and non-muscle invasive BC (NMIBC), and NMIBC was further classified into low–intermediate-risk NMIBC (LMR-NMIBC), and high-risk NMIBC (HR-NMIBC) [2, 3]. Although 70–80% of patients are diagnosed with NMIBC and among those up to 50% are LMR-NMIBC that show favorable prognosis, patients diagnosed with HR-NMIBC show increased recurrence and progression rate and poor survival once progressed to MIBC [4]. Furthermore, MIBC is aggressive with high morbidity and high risk of distant metastases [5]. Delayed diagnosis and treatment of both HR-NMIBC and MIBC have profound detrimental effects on overall survival [6].

Current gold standard for BC diagnosis remains to be cystoscopy and biopsy of suspicious lesions [7]. These costly and invasive procedures of initial diagnosis are sub-optimal with annually 20,000 cancer cases missed among moderate-to-high-risk hematuria patients, while 230,000 cases went through unnecessary cystoscopy in the USA [8]. Meanwhile, regardless the usage of multiple radiologic imaging, an estimated 10–41% of NMIBC were under-staged and required repeated transurethral resection of the bladder tumor (TURBT), possibly attributed to tumor heterogeneity and failure of detrusor muscle inclusion [9–11]. The sub-optimal diagnostic modalities and high demands of follow-ups of HR-NMIBC and MIBC patients, resulted in significant cumulative costs in BC care [12]. Non-invasive diagnostic tests with high sensitivities and/or accurate risk stratifications are therefore desirable to facilitate efficient diagnostic protocol, reduce intensive treatments from delayed diagnosis and mitigate the economic burdens.

Urine cytology as a common non-invasive test showed a high specificity for high-grade tumors but a poor sensitivity of only 17% for low-grade BC [13]. The US Food and Drug Administrations (FDA) approved urinary assays such as NMP22 (Matritech), BTA stat (Polymedco), BTA TRAK (Polymedco), and UroVysion (Abbott Molecular) reported non-inferior overall sensitivities (47–76%) and specificities (53–95%) [14]. Sensitivities for detecting low-grade and Ta tumors (39–51% and 39–53%) were still low [15]. ImmunoCyt (Scimedx) showed higher sensitivities on low-grade (74%) and Ta (73%) tumors, but the assay is of limited use as a clinical routine procedure due to high interobserver variations and extensive cost [15, 16]. Additionally, there is no test that are commercially available to identify HR-NMIBC or MIBC patients pre-surgically.

DNA methylation as one of the most common epigenetic regulatory events, has been reported to play a crucial rule in early tumorigenesis [17]. Due to the relative consistency and tissue specificity of methylation profiles between individuals and same type of tumor cells as compared to somatic mutation, DNA methylation signatures have been applied as biomarkers for non-invasive detection of carcinogenesis [18]. We have previously reported a urine DNA methylation assay by mass array (utMeMA) for early-stage, minimal, residual tumor detection and surveillance [19]. In this study, we took a previously identified set of DNA methylation markers and further developed a rapid and high-throughput urine-based PCR DNA methylation assay that measured the cancer-specific co-methylation signatures and validated the assay for clinical use of bladder cancer early detection and risk stratification.

## Results

### Bladder cancer marker characterization and binary model development

**Fig. 1:**
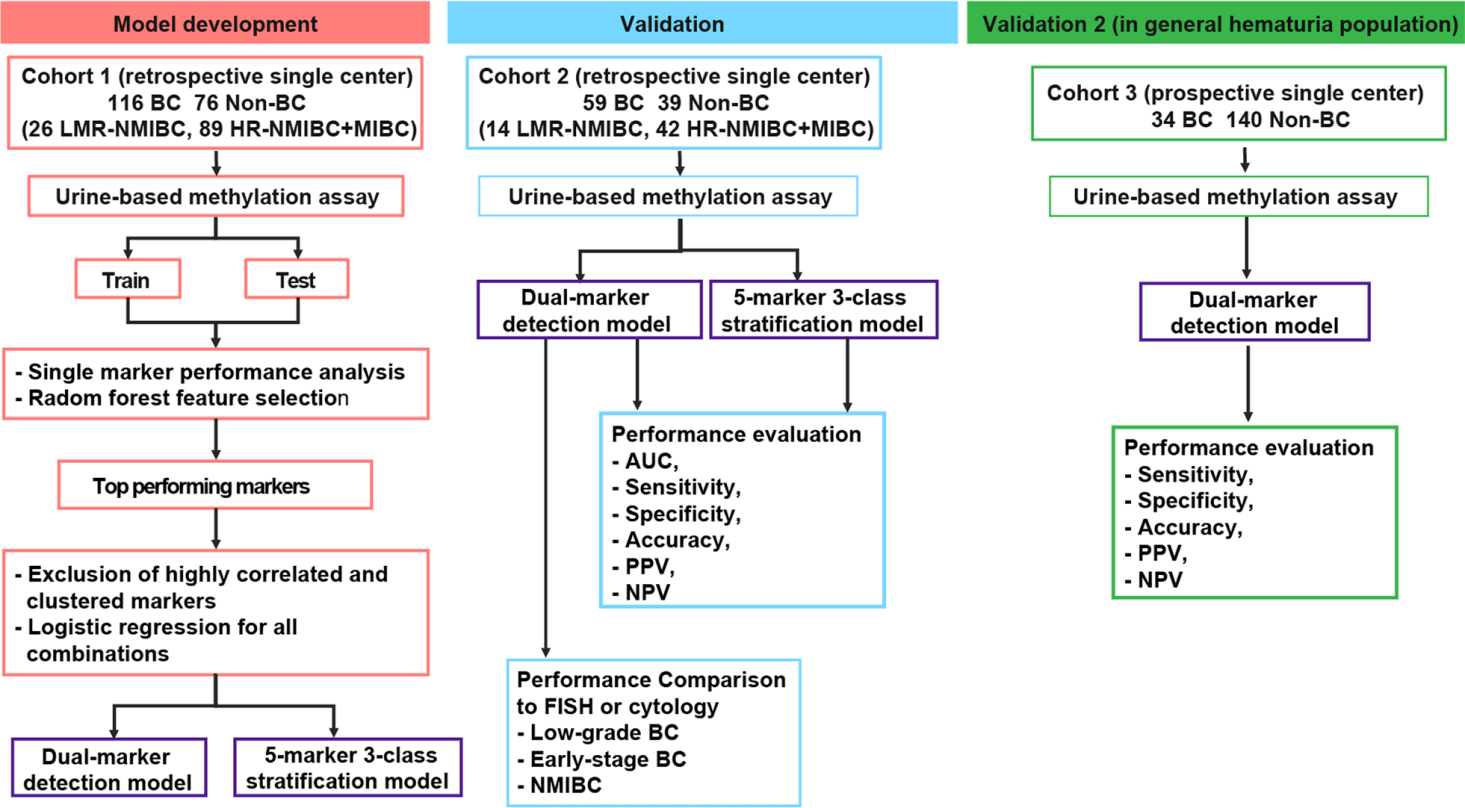
Schematic workflow of marker characterization, model development and validation for detection and risk stratification of BC

**Fig. 2:**
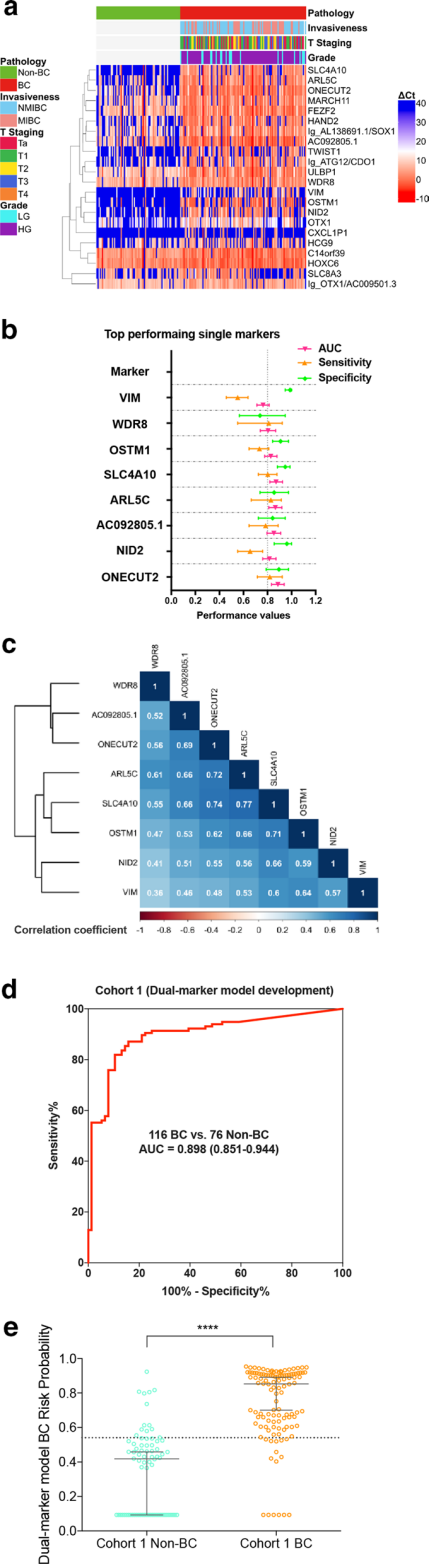
Characterization of BC DNA methylation signatures associated with BC and binary model development in Cohort 1. **a** Unsupervised hierarchical clustering of 22 DNA methylation markers; differential methylation profiles were represented reversely by ∆Ct of each marker. **b** Performance features of top individual markers for BC detection in single marker analysis; AUC, sensitivity and specificity were expressed as mean with 95% CI in 2000 bootstrap samplings; groups of clustering were sorted from unsupervised hierarchical clustering. **c** Top marker correlation matrix and unsupervised hierarchical clustering. **d** ROC curves of dual-marker detection model. **e** Distributions of dual-marker model BC risk probability in non-BC and BC groups; unpaired t test was used to analyze statistical significance; ****p < 0.0001

A urine-based PCR DNA methylation assay with a 22-marker panel based on our previous study of DNA methylation markers in BC [19] was developed, and a two-stage strategy was used to evaluate the performance of the assay, in which the markers and models were identified and finalized in Cohort 1 with 192 urine samples and subsequently validated by using two different cohorts (Cohorts 2 and 3) of 98 and 174 urine samples, respectively. The analytical workflow for marker and model development is illustrated in Fig. 1. In Cohort 1, an unsupervised hierarchical clustering of 22 markers revealed an overall higher methylation levels in the BC group than in the non-BC groups, with ∆Ct values reversely representing the co-methylation levels (Fig. 2a). Single marker analysis and random forest modeling were applied to identify the top markers for BC detection. In single marker analysis, WDR8, SLC4A10, ARL5C, AC092805.1 and ONECUT2 showed relatively high detection sensitivities of 80.2–82.8% and VIM, OSTM1, SLC4A10 and NID2 revealed relatively high specificities of 90.8–98.7% (Fig. 2b). SLC4A10, ARL5C, AC092805.1 and ONECUT2 achieved relatively high diagnostic accuracy with mean area under curves (AUC) of 0.852–0.886 (Fig. 2b). SLC4A10, ARL5C, AC092805.1 and ONECUT2, selected from random forest modeling top features, were overlapped top markers from single marker analysis (Additional file 1: Figure S1). Several pairs of the top markers (SLC4A10-ARL5C, SLC4A10-ONECUT2, ARL5C-ONECUT2 and OSTM1-SLC4A10) had high correlations (Spearman's correlation coefficient > 0.7, Fig. 2c), suggesting a potential of minimizing the number of markers for model development. By removal of highly correlated markers and iterative combination analysis of the selected top markers, a detection model consisting of 2 markers, ONECUT2 and VIM was developed. This model showed 85.4% accuracy, 87.1% sensitivity and 82.9% specificity with an AUC of 0.898 in Cohort 1 (Fig. 2d, Table 1). The risk probabilities of the BC groups derived from the model were distinctively higher than the non-BC groups (Fig. 2e).

**Table 1:** Performance features of dual-marker detection model

Tests	Sensitivity (%)	Specificity (%)	Accuracy (%)	Prevalence (%)	PPV (%)	NPV (%)
*Cohort 1 (model development)*						
Dual marker	87.1	82.9	85.4	60.4	88.6	80.8
*Cohort 2 (validation)*						
Dual marker	88.1	89.7	88.8	60.2	92.9	83.3
*Cohort 3 (validation 2-in general hematuria population)*						
Dual marker	91.2	85.7	86.8	19.5	60.8	97.6

*FISH* fluorescence in situ hybridization, *NPV* negative predictive values, *PPV* positive predictive values

### Binary model validation for detection of BC

**Fig. 3:**
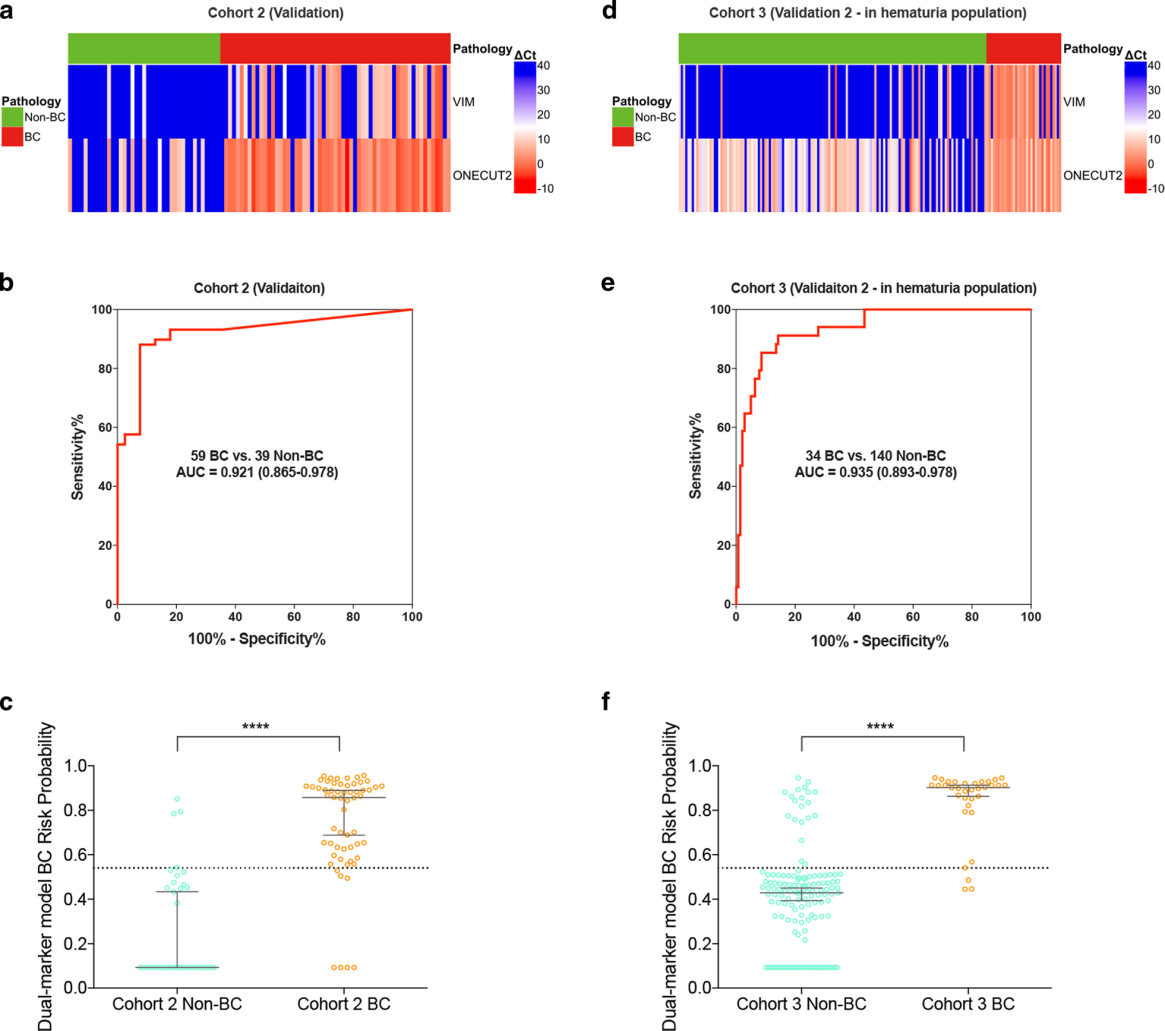
Validation of the dual-marker detection model. **a** Differential methylation levels of the dual markers between non-BC and BC groups in Cohort 2; the methylation levels were represented reversely by ∆Ct of each marker; the predicted status was made by the assay of dual-marker model. **b** ROC curves of dual-marker detection model in Cohort 2. **c** Distributions of dual-marker model BC risk probability between non-BC and BC groups in Cohort 2; unpaired t test was used to analyze statistical significance; ****p < 0.0001. **d** Differential methylation levels of the dual markers between Non-BC and BC groups in Cohort 3; the methylation levels were represented reversely by ∆Ct of each marker; the predicted status was made by the assay of dual-marker model. **e** ROC curves of dual-marker detection model in Cohort 3. **f** Distributions of dual-marker model BC risk probability between non-BC and BC groups in Cohort 3; unpaired t test was used to analyze statistical significance; ****p < 0.0001

The dual-marker detection model was further validated in two cohorts of different patient inclusion criteria for its potential clinical applications. In Cohort 2 of suspected BC populations (59 BC cases and 39 Non-BC cases), methylation levels of the two markers were higher in the BC group compared to the non-BC group, and the predicted status of the two groups was highly consistent with pathology determination of the samples (Fig. 3a). Consistent with Cohort 1, the model showed 88.8% accuracy, 88.1% sensitivities, 89.7% specificities and an AUC of 0.921 in this cohort (Fig. 3b, Table 1). BC risk probabilities of the BC group were significantly higher than those in the non-BC groups under the model (Fig. 3c). Additionally, the model was validated with another prospective cohort (Cohort 3) to evaluate the capability of the assay for ruling out non-BC patients from excessive cystoscopy in hematuria populations. Methylation levels of the two markers showed similar distinguishing capacities of non-BC and BC groups as compared to Cohorts 1 and 2 (Fig. 3d). The model also demonstrated a consistently high accuracy (86.8%), sensitivity (91.2%) and specificity (85.7%) with an AUC of 0.935 in Cohort 3, with risk probabilities of the BC group significantly higher than the non-BC groups (Table 1, Fig. 3e, f). Collectively, these data indicated that the assay of the dual-marker model showed high sensitivity and strong diagnostic power in the detection of BC.

### Assay of dual-marker model features superior sensitivity to urine cytology and FISH tests

**Fig. 4:**
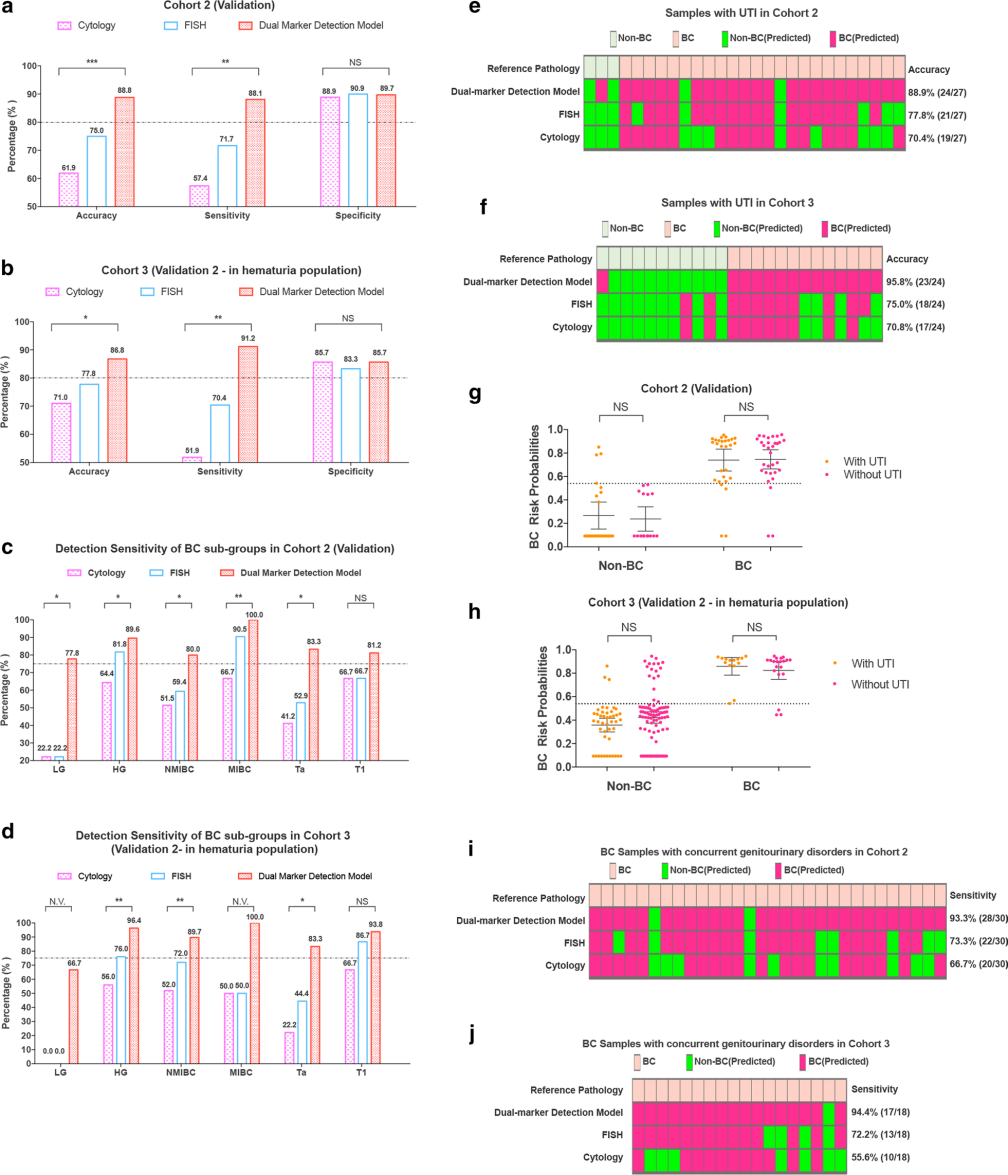
Performance of dual-marker detection model compared to cytology and FISH in Cohorts 2 and 3. **a**, **b** Diagnostic accuracy, sensitivity and specificity of dual-marker model compared to cytology and FISH test in Cohorts 2 (**a**) and 3 (**b**), respectively; statistical significance of the three tests was assessed by *χ*^2^ test; **p < 0.01; ***p < 0.001. **c**, **d** Detection sensitivity of BC sub-groups by dual-marker model compared to cytology and FISH in Cohorts 2 (**c**) and 3 (**d**), respectively; statistical significance of the three tests was assessed by *χ*^2^ test; *p < 0.05. **e**, **f** Accuracy of the dual-marker detection model compared to FISH and cytology in samples with urinary tract infections in Cohorts 2 and 3, respectively. **g**, **h** Distributions of BC risk probabilities between samples with UTI and without UTI in non-BC and BC patients in Cohorts 2 and 3, respectively; each dot represented one sample, and gray lines indicated corresponding mean and 95% confident intervals in each group; the dashed line indicated the model cutoff; unpaired t test was used to analyze statistical significance. **i**, **j** Sensitivity of the dual-marker detection model compared to FISH and cytology in BC sample with concurrent genitourinary disorders in Cohorts 2 and 3, respectively

We further compared performance of the assay of dual-marker model to cytology and FISH tests in the two validation cohorts for BC detection. The assay exhibited superior overall diagnostic accuracy (88.8% vs. 61.9% by cytology and 75.0% by FISH) and sensitivity (88.1% vs. 57.4% by cytology and 71.7% by FISH), with a similar specificity (89.7% vs. 88.9% by cytology and 90.9% by FISH) in Cohort 2 (Fig. 4a). Consistently, the assay out-performed cytology and FISH tests on diagnostic accuracy (86.8% vs. 71.0% and 77.8%) and sensitivity (91.2% vs. 51.9% and 70.4%) with comparable specificity (85.7% vs. 85.7% and 83.3%) in Cohort 3 (Fig. 4b). In particular, the assay showed significantly higher sensitivities than cytology and FISH for low-grade tumor (66.7–77.8% vs. 0.0–22.2% and 0.0–22.2%), NMIBC (80.0–89.7% vs. 51.5–52.0% and 59.4–72.0%) and Ta tumor detection (83.3% vs. 22.2–41.2% and 44.4–52.9%) in both cohorts (Fig. 4c, d).

With regard to samples from patients with concurrent genitourinary disorder such as urinary tract infection (UTI), in which excessive granulocytes and massive bacteriuria in specimen may impair diagnostic capacity [20, 21], the assay showed higher accuracies (88.9% and 95.8%) than cytology (70.4% and 70.8%) and FISH (77.8% and 75.0%) in Cohorts 2 and 3 (Fig. 4e, f). Comparisons of samples with UTI and without UTI indicated no significant differences on the model risk probabilities in the two cohorts, in both non-BC and BC groups, further indicating UTI conditions did not interference with the DNA methylation assay (Fig. 4g, h). In addition, the assay specificities in patients diagnosed with UTI, bladder benign lesions (BBL), benign prostatic hyperplasia (BPH), urolithiasis and other disorders did not show significant differences (Additional file 2: Figure S2). In the BC cases with concurrent genitourinary disorders including benign prostatic hyperplasia, atypia and inflammatory bladder benign lesions, neoplastic lesions and UTI, the assay also exhibited higher sensitivities (93.3% and 94.4%) than cytology (66.7% and 55.6%) and FISH (73.3% and 72.2%) in Cohorts 2 and 3 (Fig. 4i, j). These findings indicated that the assay of dual-marker model has superior capacities in diagnosing cases with concurrent diseases as compared to cytology and FISH.

### Model development and validation for three-class BC risk stratifications

**Fig. 5:**
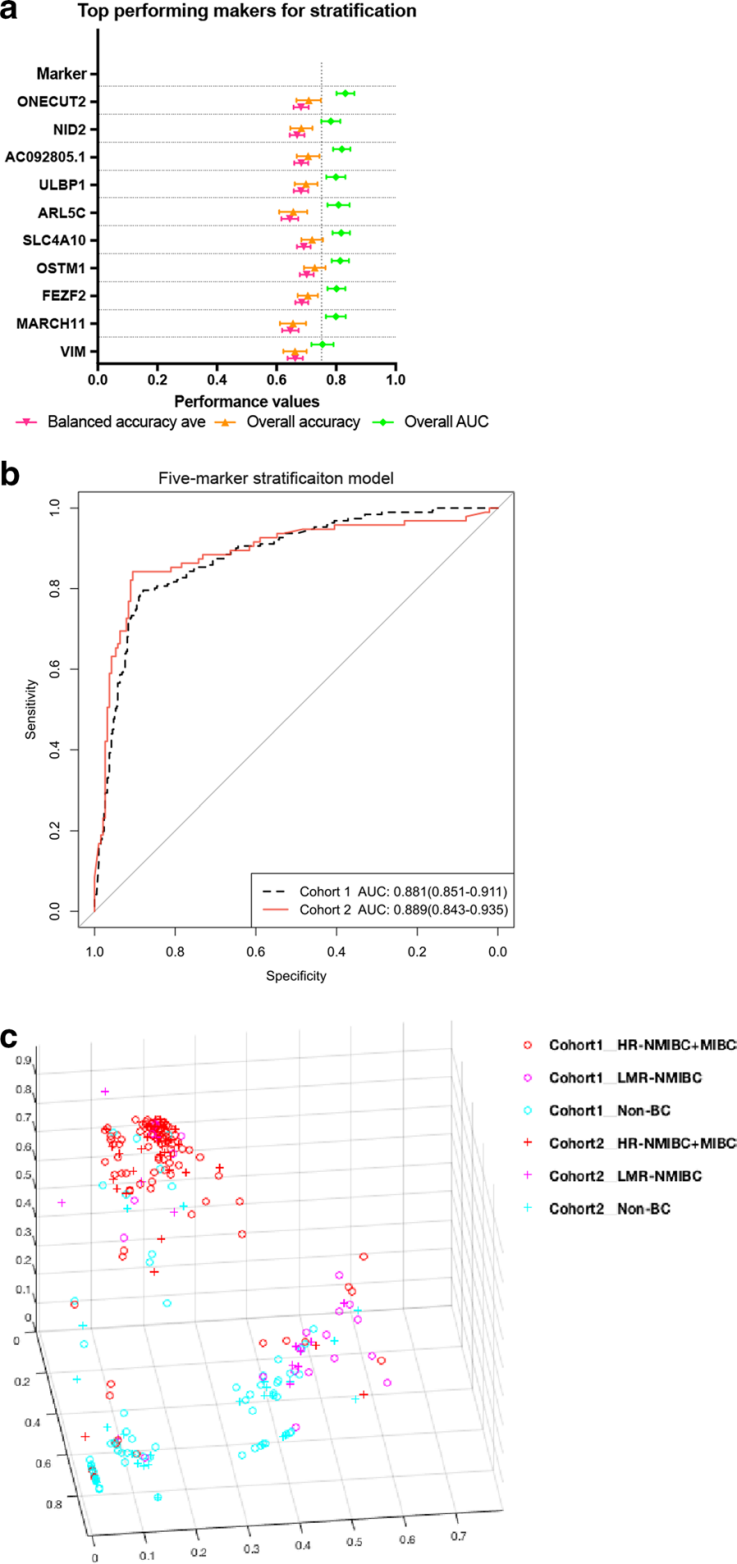
A five-marker three-class stratification model for bladder cancer risk stratification. **a** Performance features of top individual markers for risk stratification in Cohort 1 from single marker analysis; average of balanced accuracies of the three groups (where balanced accuracy was calculated as ½ of sum of sensitivity and specificity of each group), overall AUCs (where AUC referred to area under ROC generated by sensitivity and specificity summing up of true positive, false positive, true negative and false negative of each class based on the micro-average method) and overall accuracies were expressed as mean with 95% CI in 100 splits of train-test sampling. **b** ROC curves of stratification model in Cohorts 1 and 2; AUC referred to area under ROC generated by sensitivity and specificity summing up of true positive, false positive, true negative and false negative of each class based on the micro-average method; **c** distributions of the three-class probabilities from the stratification model for samples in Cohorts 1 and 2; each sample was depicted by the three coordinates representing the probabilities of the respective non-BC, LMR-NMIBC or HR-NMIBC + MIBC group

To gain more insights on BC risk classification, methylation profiles of the 22 markers in Cohort 1 were explored for discriminating the following three groups: cases of non-BC, HR-NMIBC + MIBC and LMR-NMIBC, respectively. Risk levels of NMIBC were determined according to AUA definition and NCCN guideline [9]. The single marker analysis revealed that FEZF2, OSTM1, SLC4A10, ARL5C, AC092805.1, ONECUT2, VIM, ULBP1 and NID2 had relatively high overall AUCs, average balanced accuracies or overall accuracies (Fig. 5a). OSTM1, SLC4A10, ULBP1, AC092805.1, NID2 and ONECUT2 also appeared to be top features for the three classification in random forest modeling (Additional file 3: Figure S3). With comparison of multiple performance features in iterative combinations of the selected top markers, a model with VIM, OSTM1, SLC4A10, AC092805.1 and ONECUT2 showed an overall AUC of 0.881 and accuracy of 78.0% in Cohort 1 and was validated with consistent AUC of 0.889 and accuracy of 82.1% in Cohort 2 (Fig. 5b, Table 2). Based on distributions of three-class probabilities from the stratification model, samples of the three groups were clustered separately from each other (Fig. 5c). Importantly, the model identified non-BC group with 87.2% sensitivity and 91.1% specificity and identified HR-NMIBC + MIBC patients with 90.5% sensitivity and 86.8% specificity in Cohort 2 (Table 2). While HR-NMIBC + MIBC patients may have relatively higher tumor contents that can be more easily detected by urine cytology or FISH, positive rates for urine cytology and FISH for identifying these patients as BC positive were 65.0% and 82.1%, respectively (Table 3), indicating a significant proportion of patients with high-risk BC were missed by the two tests. As compared to 90.5% of the positive rates of the five-marker model for identifying these patients as HR-NMIBC + MIBC, patients in these groups missed by urine cytology or FISH tests may have better chance to be identified preclinically by the five-marker model.

**Table 2:** Performance features of five-marker stratification model

		Prediction			Sensitivity (%)	Specificity (%)	PPV (%)	NPV (%)	Balanced accuracy (%)	Overall accuracy (%)	Overall AUC
		Non-BC	LMR-NMIBC	HR-NMIBC + MIBC							
*Cohort 1 (model development)*											
Reference pathology	Non-BC	63	4	9	87.8	82.9	88.6	81.8	85.4	78.0	0.881
	LMR-NMIBC	5	12	9	46.2	93.9	54.5	91.7	70.0		
	HR-NMIBC + MIBC	9	6	74	83.1	82.4	80.4	84.8	82.7		
*Cohort 2 (validation)*											
Reference pathology	Non-BC	34	3	2	91.1	87.2	91.1	87.2	89.1	82.1	0.889
	LMR-NMIBC	3	6	5	42.9	93.8	54.5	90.5	68.3		
	HR-NMIBC + MIBC	2	2	38	90.5	86.8	84.4	92.0	88.6		

*HR-NMIBC* high-risk NMIBC, *LMR-NMIBC* low–intermediate-risk NMIBC, *MIBC* muscle invasive bladder cancer

**Table 3:** Positive rates of urine cytology and FISH for indentified HR-NMIBC + MIBC as BC

Tests	Cases with test results	Positive as HR-NMIBC + MIBC	Positive as BC	Positive rate (%)
Five-marker model	42	38	–	90.5
Cytology	40	–	26	65.0
FISH	39	–	32	82.1

**Fig. 6:**
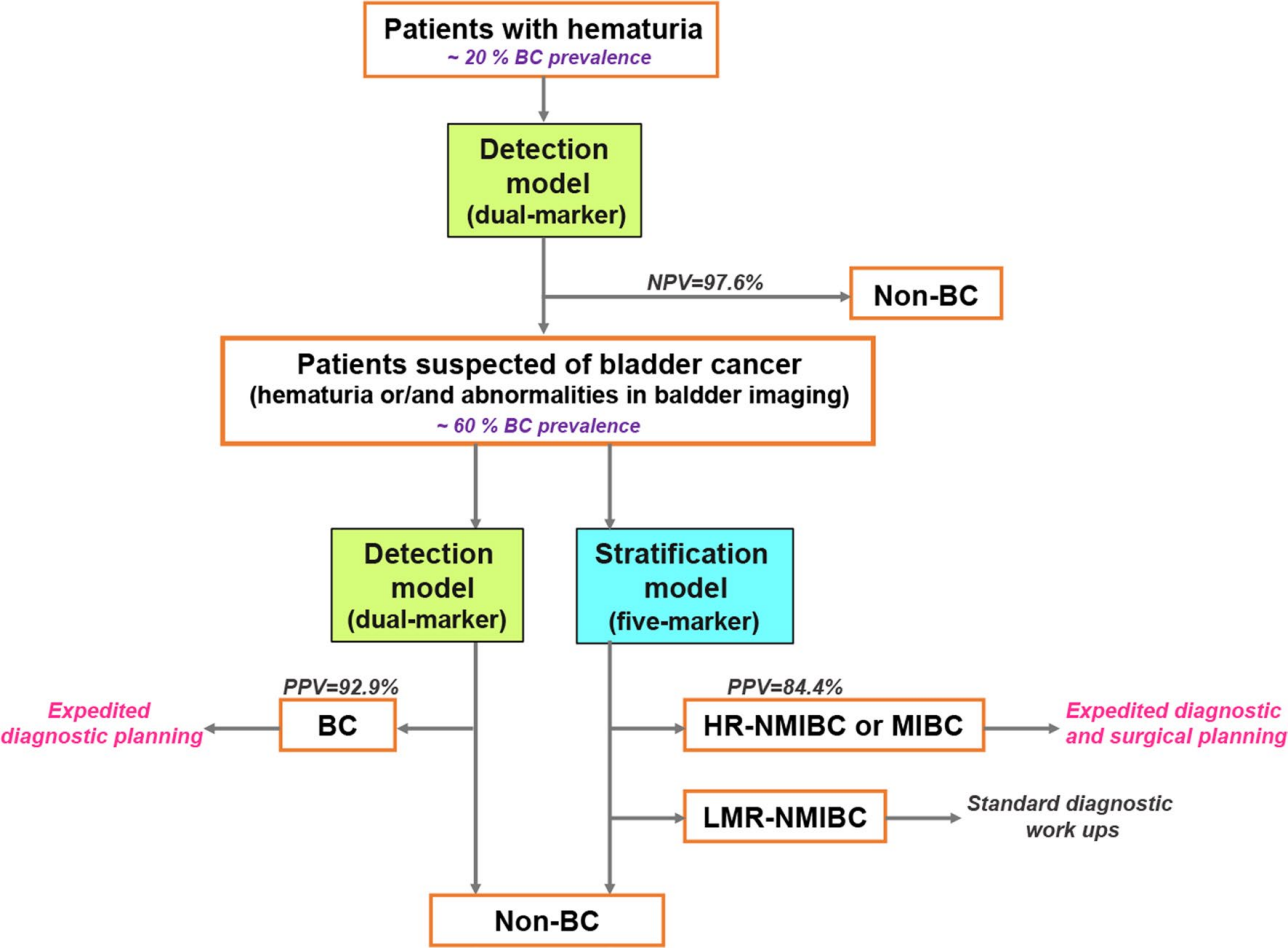
A schematic overview of proposed clinical applications of detection model or stratification model by the methylation assay for systematic bladder cancer management

Based on the clinical performance of the assay, clinical applications of assay with the detection model and the stratification model were proposed (Fig. 6). The assay with the detection model may aid in avoiding excessive cystoscopies in patients with clinical suspect of BC by an NPV of 97.6% in a general screening clinical setting (Cohort 3), where the prevalence of BC in hematuria patients was about 20%. On the other hand, in a primary confirmative diagnostic setting (Cohort 2), where patients suspected of BC have a BC prevalence of about 60%, the assay of detection model may effectively confirm BC patients with a sensitivity of 88.1% and a PPV of 92.9%, for expedited diagnostic planning and intervention planning. Furthermore, within the same clinical setting, the stratification model identified the BC patients of high risk (HR-NMIBC or MIBC) with a sensitivity of 90.5% and a PPV of 84.8% for expedited diagnostic and surgical planning.

## Discussions

In this study, we developed and validated a urine-based PCR DNA methylation assay for early detection and pre-operative risk stratification of BC. The PCR-based DNA methylation assay interrogated the cancer-specific methylation patterns consisting of multiple CpG dinucleotides and thus allowed highly sensitive and specific detection of cancer-specific methylation events of low frequency in patient samples of early-stage tumors. Consistent with our previous study [19], the dual-marker model from the PCR DNA methylation assay showed a similar overall accuracy, sensitivity and specificity for BC detection.

Interestingly, though the previously reported dual-marker model of utMeMA (utMeMA markers corresponding to methylation regions of OTX1 and intergenic region of Ig_AL138691.1/SOX1 in the 22-marker methylation panel in this study) in the mass array study was different from the dual-marker model (ONECUT2 and VIM) in this study, OTX1 and VIM clustered into one group and intergenic region of Ig_AL138691.1/SOX1 and ONECUT2 clustered into another group in the methylation profiling (Fig. 2a). The observation indicated the high correlations of different markers in the same clusters, which may have converged into two signal regulation pathways contributing to BC tumorigenesis. The same marker clusters identified by both the utMeMA and the PCR DNA methylation assay highlighted the consistencies of the underlined pathways represented by the two assays.

For the BC detection model validation, we included two separate cohorts with different patients inclusion criteria to evaluate the performance of the assay under different clinical settings. Patients suspected of BC recruited in Cohort 2 usually showed abnormalities in urological imaging in addition to hematuria which required further confirmative diagnosis. The assay of dual-marker model showed 92.9% PPV and 10.3% false-positive rate at a primary confirmative diagnostic setting (60.6% BC prevalence, Cohort 2), allowing informative inclusion of BC patients without causing excessive invasive examination due to false-positive cases. On the other hand, while patients suspected of BC required additional examination from the clinicians or imaging, the assay can be applied in hematuria populations (Cohort 3) to potentially aid in a diagnostic work-up decision. Thus, the assay was further validated in a second cohort (Cohort 3) in a more general screening setting with about 20% BC prevalence, which is consistent with the reported BC prevalence in hematuria population [22]. The high NPV (97.6%) of the assay further implicated a potential application of the assay for clearing patient of low cancer risk from excessive cystoscopy. The potential clinical utility of the assay may need further evaluation as compared to standard work-ups by multi-center prospective studies.

Compared to other reported methylation assays, the assay performance of detection model of ONECUT2 and VIM (88.1–91.2% sensitivity and 85.7–89.7% specificity) was superior than those with NID2 and TWIST1 (76.2% sensitivity and 83.3% specificity), BCL2, CDKN2A and NID2 (80.9% sensitivity and 86.4% specificity) and HOXA9, PCDH17, POU4F2 and ONECUT2 (90.5% sensitivity and 73.2% specificity) [23–25]. Some of the methylation sites and their adjacent ones of the published methylation markers, such as NID2 and TWIST1, were also included in the 22-marker panel. As compared to the dual-marker model of ONECUT2 and VIM, the individual marker (NID2 or TWIST1) or the combined model showed inferior AUCs in cohort 2 (0.921 vs. 0.793, 0.796 and 0.842) (Additional file 4: Figure S4). Performance of NID2 in our methylation assay showed 65.5% sensitivity and 96.1% specificity in Cohort 1 for detecting BC (Fig. 2b), which was consistent with the previous reports (61.9% sensitivity and 90.9% specificity) [24]. The AUCs of NID2 and TWIST1 in cohort 2 were also consistent with the reported AUCs of 0.781 and 0.830 [23]. These observations suggested conceptual and experimental advantages of the dual-marker model in clinical utility over published methylation markers.

Compared to other non-invasive tests commonly used in the clinic and those approved by US FDA, including NMP22, BTA stat, BTA TRAK and UroVysion, the dual-marker assay showed superior sensitivities in detecting low grade (66.7–77.8% by the assay versus 0–22.2% by cytology and FISH, 39–51% by the four US FDA-approved assays and 74% by ImmunoCyt) and Ta tumors (83.3% by the assay vs. 22.2–41.2% of cytology, 44.4–52.9% by FISH, 39–51% by the four US FDA-approved assays and 73% by ImmunoCyt) [15]. The superior sensitivity of the assay for detecting BC may be due to the technical advantages of using multiple BC-specific methylation markers, which may improve the detection threshold in early-stage BC and the signal stability from enriched urine genomic DNA. While the NMP22, BTA stat and BTA TRAK are protein-based tests in which the targets of interest are prone to degradation and less specific, the UroVysion requires fresh urine samples, and results were affected by the integrity of cells within the urine. The sensitivity of detecting Ta tumor was also superior to the reported methylation assays by DLX1 and ITGA4 (50.0–84.6%) and BCL2, CDKN2A and NID2 (61.1%) [24, 26]. These features may allow for early BC diagnosis and enable favorable treatment and surveillance modalities. It is noticeable that the proportion of early-stage BC patients (Ta and T1/NMIBC) in Cohort 3 was higher as compared to Cohorts 1 and 2, implying that standard work-ups for identifying suspected BC patients may miss some of the patients with early-stage tumors. Application of the assay in a general screening setting may help for identifying these early-stage patients.

In addition to binary classification of BC and non-BC patients, the assay with a five-marker three-class risk stratification model was also developed to classify suspected BC patients as non-BC, LMR-NMIBC or HR-NMIBC + MIBC groups before cystoscopy or TURBT. The desirable sensitivities (90.5%), specificities (86.8%) and PPV (84.4%) in classifying HR-NMIBC + MIBC patients ensured a pre-operative accurate identification and reduced possible missed diagnosis by urine cytology or FISH. As compared to current standard work-ups, the pre-operative risk stratification may provide additional information and guide diagnostic and surgical planning, such as inclusion of muscularis propria and random sampling [27].

The dual-marker assay (sensitivities of 88.1–91.2% and specificities of 85.7–89.7%) also exhibited comparable performance to the methylation assay currently in the Europe’s study, the Bladder EpiCheck, with reported sensitivities of 62.5–90% and specificities of 82.1–90.0% [28]. While the Bladder EpiCheck utilizes a panel of 15 methylation markers, the assay of dual-marker and five-marker three-class risk stratification models can further reduce the cost in a clinical screening setting. Complementary to the application of the Bladder EpiCheck in the recurrent surveillance, our assay, which can screen out non-BC patients from the general hematuria group and preclinically identify early or HR-NMIBC and MIBC in initial diagnosis, may work in association with the Bladder EpiCheck in BC patients’ follow-up for an integrated diagnostic management.

In terms of test interference and sample collection, unlike the urine cytology and FISH, in which the test accuracy may be affected in patients with concurrent genitourinary disorders and samples must be freshly collected and analyzed, the methylation assay showed higher diagnostic accuracy in these patients as compared to cytology and FISH and was not affected by UTI. Additionally, with the association of the at-home urine self-collection device and the stability of urine samples, the assay may reduce hospital visits of diagnostic cystoscopies and urine cytology for patients and laboratory work with fresh urines for cytology technicians for the prevention of viral spread in the COVID-19 era.

## Conclusions

The urine-based DNA methylation assay with both the dual-marker detection model and the five-marker risk stratification model demonstrated a clinically feasible test for non-invasive systematic diagnosis of BC.

## Methods

### Study design

Retrospective single-center cohorts were designed to develop (Cohort 1) and validate (Cohort 2) the performance of a DNA methylation assay. Patients suspected of having BC (with hematuria and/or primary urological imaging abnormalities [29, 30], but with no history of malignancies) were recruited from June 2017 to December 2019 sequentially in Sun Yat-Sen Memorial Hospital. Positive BC cases were confirmed by pathology determination of either cystoscopy or surgical pathology. Non-BC group included patients diagnosed with urological calculus, urinary tract infections (UTI) and genitourinary benign lesions, as determined by the final diagnostic results. Urine samples collected during the recruitment period were randomly divided into Cohort 1 (*n* = 192, 116 BC cases and 76 non-BC controls) and 2 (*n* = 98, 59 BC cases and 39 non-BC controls) by a bio-informatician blinded to test results of the methylation assay and tested for the methylation assay by researchers who were blinded to the cohort division and any clinical information of the patients. In addition, only after the models and the assay cutoffs were finalized, the clinical outcome information of Cohort 2 was released for validation analysis. Researchers for patient data curation who reviewed and ensured the data merging and completeness were blinded to model development and validation analysis. Patient characteristics are summarized in Additional file 5: Table S1. Some patients in the two cohorts also underwent urinary cytology and fluorescence in situ hybridization (FISH) test as followed by standard of care for diagnosing BC in China and urologists’ instructions.

In a prospective single-center study for validation of the assay for BC detection (Cohort 3), patients with general hematuria, currently not diagnosed with concurrent non-genitourinary malignancies and with no history of genitourinary malignancies were recruited from December 2019 to August 2020 in Sun Yat-Sen Memorial Hospital (*n* = 174, 34 BC cases and 140 non-BC controls). Positive BC cases were confirmed by either cystoscopy or surgical pathology. Non-BC group included patients diagnosed with urological calculus, UTI, genitourinary benign lesions and other genitourinary malignancies such as prostate cancer, renal cancer and small cell neuroendocrine carcinoma as determined by the final diagnostic results. The patient characteristics are summarized in Additional file 5: Table S2.

### Urine sample collection and processing

Urine samples from all patients were collected only once and prior to cystoscopy or surgery in all the three cohorts for the methylation assay. A total of 200 ml urine was collected for each patient when 100 ml of the urine was used for cytology and FISH test if patients were required by the urologists for these tests. The final diagnostic results of non-BC patients under the same visit for methylation assay were used. In total, 100–200 ml of urine from each patient was collected in a urine collection device, U-do (AnchorDx, China, Catalog No. U0021) containing the urine preservatives for the methylation assay. Urine specimens were stored at 2–8 °C within 5 days before processing. Cell debris and pellets from the urine specimens were obtained by centrifugation at 3000 g for 10 min followed by a wash of phosphate-buffered saline (PBS) and immediately stored at − 80 °C before DNA extraction.

### DNA extraction, bisulfite treatment and methylation analysis

Genomic DNA from cell debris and pellets of the urine specimens was extracted using the QIAamp DNA Blood Mini Kit (Qiagen, Germany, Catalog No. 51106) following manufacturer’s instruction and quantified by the Qubit Assay (Thermo Fisher Scientific, USA, Catalog No. Q32851). Briefly, samples were lysed with Buffer AL and protease K at 56 °C for 10 min, and DNA from the lysate was precipitated with ethanol and purified with the QIAamp Mini spin column by washing with 500 μl Buffer AW1 and 500 μl Buffer AW2 subsequently. The column was centrifuged and DNA was eluted with Buffer AE for quantification. Samples of genomic DNA of less than 25 ng were excluded from the assay due to insufficient materials for the assay.

Bisulfite treatment was then carried out on 25 ng of genomic DNA of each sample with the EZ-96-DNA Methylation-Direct MagPrep Kit (Zymo Research, USA, Catalog No. D5044) following instructions for purified DNA as starting materials. In total, 20 μl DNA was treated with 130 μl of CT Conversion Reagent in a thermal cycler (Thermo Fisher, USA, Catalog No. 4484073) at 98 °C for 8 min and 64 °C for 3.5 h. The treated DNA was then purified by 10 μl MagBinding Beads with M-Wash Buffer. In total, 200 μl of M-Desulfonation Buffer was added to the beads for an incubation of 15 min at room temperature (20–30 °C). DNA after desulfonation was further purified by washing with the M-Wash Buffer twice and eluted with 20 μl of M-Elution Buffer.

The methylation of bisulfite-treated DNA was analyzed by a 22-marker BC DNA methylation panel designed based on our previous study of DNA methylation markers in BC [19] (Additional file 5: Table S3) (AnchorDx, China, Catalog No. UME043) on the QuantStudio 3 Real-Time PCR System (Thermo Fisher, USA). EpiTect PCR Control DNA Set (Qiagen, Germany, Catalog No. 59695) was served as positive and negative controls. Briefly, the 22-marker targeted pre-amplification of methylated regions was carried out on 15 μl of the bisulfite-treated DNA using 25 μl of the Meth-Pre-Amp Master Mix (AnchorDx, China, Catalog No. UME043-01) and 10 μl of Meth-Pre-Amp 22-Marker BC Panel (AnchorDx, China, Catalog No. UME043-02) in the kit in a thermal cycler (Thermo Fisher, USA, Catalog No. 4484073) at 98 °C for 30 s, 20 cycles at 98 °C for 15 s, 60 °C for 15 s and 72 °C for 15 s, and 72 °C for 5 min. The amplified products were further quantified by multiplex quantitative PCR using the Meth-Quant Master Mix (AnchorDx, China, Catalog No. UME043-03) and the 22-marker BC Detect Panel (AnchorDx, China, Catalog No. UME043-04). Briefly, 1 μl of the amplified products was used in a reaction for quantifying two or three targets of interest with 7 μl of Meth-Quant Master Mix and 2 μl of the 22-marker BC Detect Panel. The reactions were carried out in a real-time PCR system at 95 °C for 5 min, 40 cycles at 95 °C for 15 s and 62 °C for 40 s, with fluorescent signals collected at the annealing/extension step (62 °C for 40 s). Representative amplification curves of selected regions of interest and region of internal control in a randomly selected case, positive control, negative control and no template control (NTC) are shown in Additional file 6: Figure S5.

### Data and statistical analysis

Co-methylation levels of a genomic region of interest were expressed by ∆Ct (cycle threshold), where ∆Ct = Mean Ct (region of interest)—Mean Ct (region of internal control). The methylated bisulfite-converted DNA fragments of regions of interest were amplified by the assay, while a DNA fragment of ATCB in the absence of methylation sites was amplified as a control for total bisulfite-converted DNA measurement. The ∆CT values were inversely correlated with the percentages of methylated molecules among total bisulfite-converted DNA molecules. ∆Cts of 35 were applied for target regions with undetermined Ct values. R packages of ComplexHeatmap, Ape and Corrplot were used for unsupervised hierarchical clustering and correlation analysis. Performance of individual and combinations of markers were analyzed using R pROC package with 2000 bootstraps. The sensitivities and specificities were calculated for individual markers with each bootstrap when the cutoffs were set as maximum values of the Youden’s index. The original 4-group categories by urinary cytology were combined into two groups, with suspicious and positive cases considered as positive and atypical and negative cases considered as negative [31]. Random forest-based analysis and logistic regression-based model constructions were conducted using Python Sklearn packages. For binary classification, logistic regression was modeled based on the ∆Ct values of individual markers as compared to the patients’ diagnostic results. For three-class classification, logistic regression based on a one-vs.-rest strategy was used to build binary classifiers for each class to determine the likelihoods of a sample belonging to a specific class, and the highest score among the three probabilities for the three classes determined the classification of the sample.

## Supplementary Information

**Additional file 1: Figure S1**. Feature importance of the 22 markers in random forest modeling in Cohort 1 for BC detection; random forest modeling was performed in 100 splits of train-test sampling in the cohorts and contributions of each markers on each of the models were scored as feature importance; the importance of each marker for classifying BC and non-BC cases was expressed as mean with range in 100 splits of train-test sampling.

**Additional file 2: Figure S2**. Specificities of the assay of dual-marker model in patients with different disorder conditions in cohorts 2 and 3. Statistical significance was assessed by χ^2^ test; NS, no statistical significance.

**Additional file 3: Figure S3**. Feature importance of the 22 markers in random forest modeling in Cohort 1 for risk stratification of BC; random forest modeling was performed in 100 splits of train-test sampling in the cohorts and contributions of each markers on each of the models were scored as feature importance; the importance of each marker for classifying non-BC, LMR-NMIBC and HR-NMIBC+MIBC cases was expressed as mean with range in 100 splits of sampling.

**Additional file 4: Figure S4**. ROC curves of NID2, TWIST1 and model of combinations of NID2 and TWIST1 for detection of BC in cohort 2. BBL, bladder benign lesions; BPH, benign prostatic hyperplasia; UTI, urinary tract infections.

**Additional file 5**. Supplementary information.

**Additional file 6: Figure S5**. Amplification curves of selected markers and internal controls in the methylation assay. Amplification signals were expressed as ∆Rn, where Rn was the target fluorescent signal normalized to the signal of the passive reference dye and ∆Rn was the Rn value minus that of the instrument baseline signal. **A**, Amplification curves of a methylated bisulfite-converted DNA fragment of ONECUT2 in a randomly selected case (blue lines), positive control (orange lines), negative control (green lines) and NTC control (gray line); **B**, amplification curves of a methylated bisulfite-converted DNA fragment of VIM in a randomly selected case (blue lines), positive control (orange lines), negative control (green lines) and NTC control (gray line); **C**, amplification curves of a DNA fragment of ATCB as a control for the measurement of total bisulfite-treated methylated and unmethylated DNA molecules in a randomly selected case (blue lines), positive control (orange lines), negative control (green lines) and NTC control (gray line).

## Data Availability

The datasets used and/or analyzed during the current study are available from the corresponding author on reasonable request.

## Abbreviations

AUC: Area under curve
BC: Bladder cancer
BLCA: Bladder urothelial carcinoma
BBL: Bladder benign lesions
BPH: Benign prostatic hyperplasia
Ct: Cycle threshold
FDA: Food and Drug Administrations
FISH: Fluorescence in situ hybridization
HR-NMIBC: High-risk NMIBC
LMR-NMIBC: Low–intermediate-risk NMIBC
NMIBC: Non-muscle invasive bladder cancer
MIBC: Muscle invasive bladder cancer
NPV: Negative predictive values
NTC: No template control
PBS: Phosphate-buffered saline
PPV: Positive predictive values
ROC: Receiver operating characteristic
TURBT: Transurethral resection of the bladder tumor
TCGA: The Cancer Genome Atlas
UTI: Urinary tract infections
