# Supplementary figures and images for: A urine-based DNA methylation assay to facilitate early detection and risk stratification of bladder cancer
Source: Clin Epigenetics. 2021 Apr 26;13:91. doi: 10.1186/s13148-021-01073-x (PMC8072728; doi:10.1186/s13148-021-01073-x)

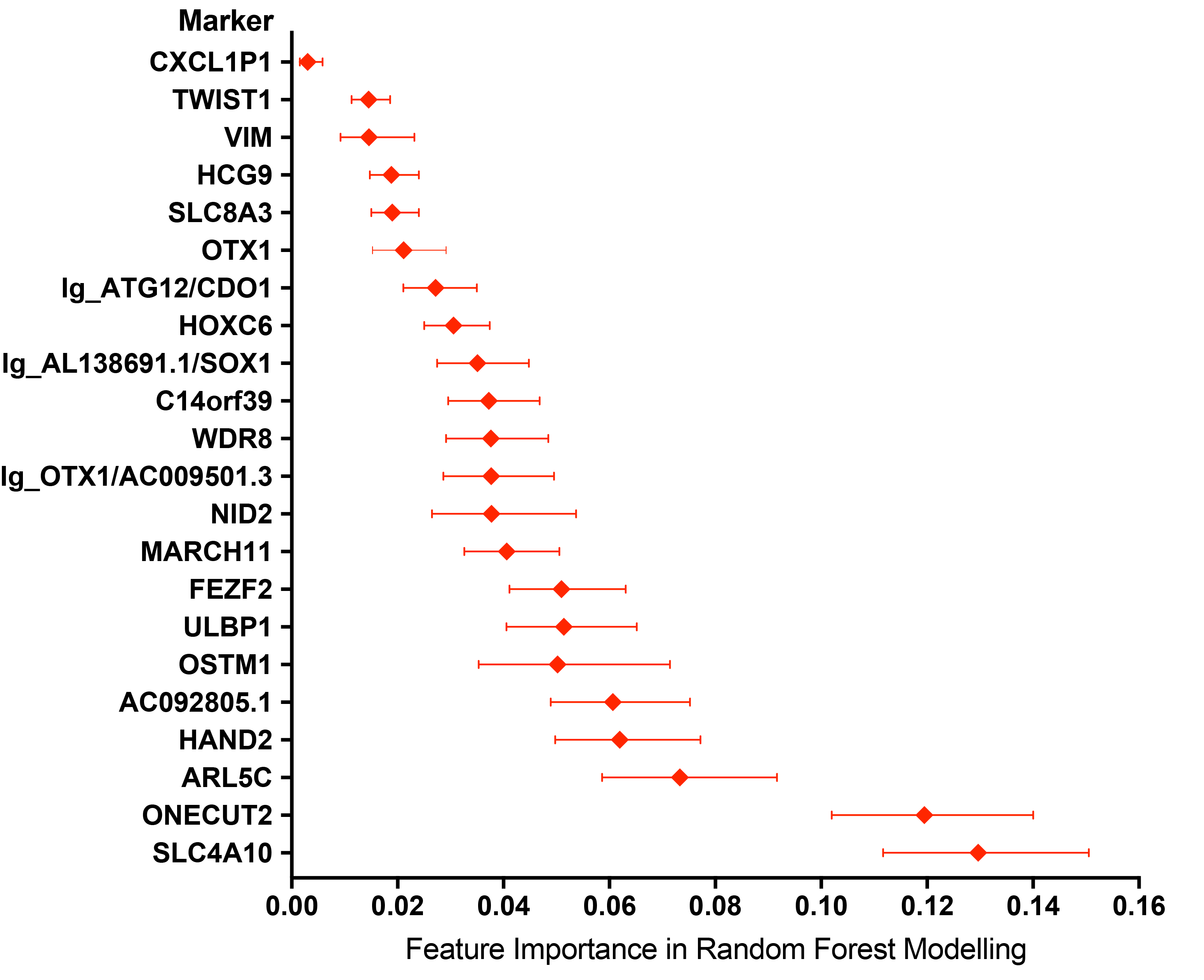

Supplement: Supplementary file 1 — Additional file 1: Figure S1. Feature importance of the 22 markers in random forest modeling in Cohort 1 for BC detection; random forest modeling was performed in 100 splits of train-test sampling in the cohorts and contributions of each markers on each of the models were scored as feature importance; the importance of each marker for classifying BC and non-BC cases was expressed as mean with range in 100 splits of train-test sampling. [file 13148_2021_1073_MOESM1_ESM.tif]

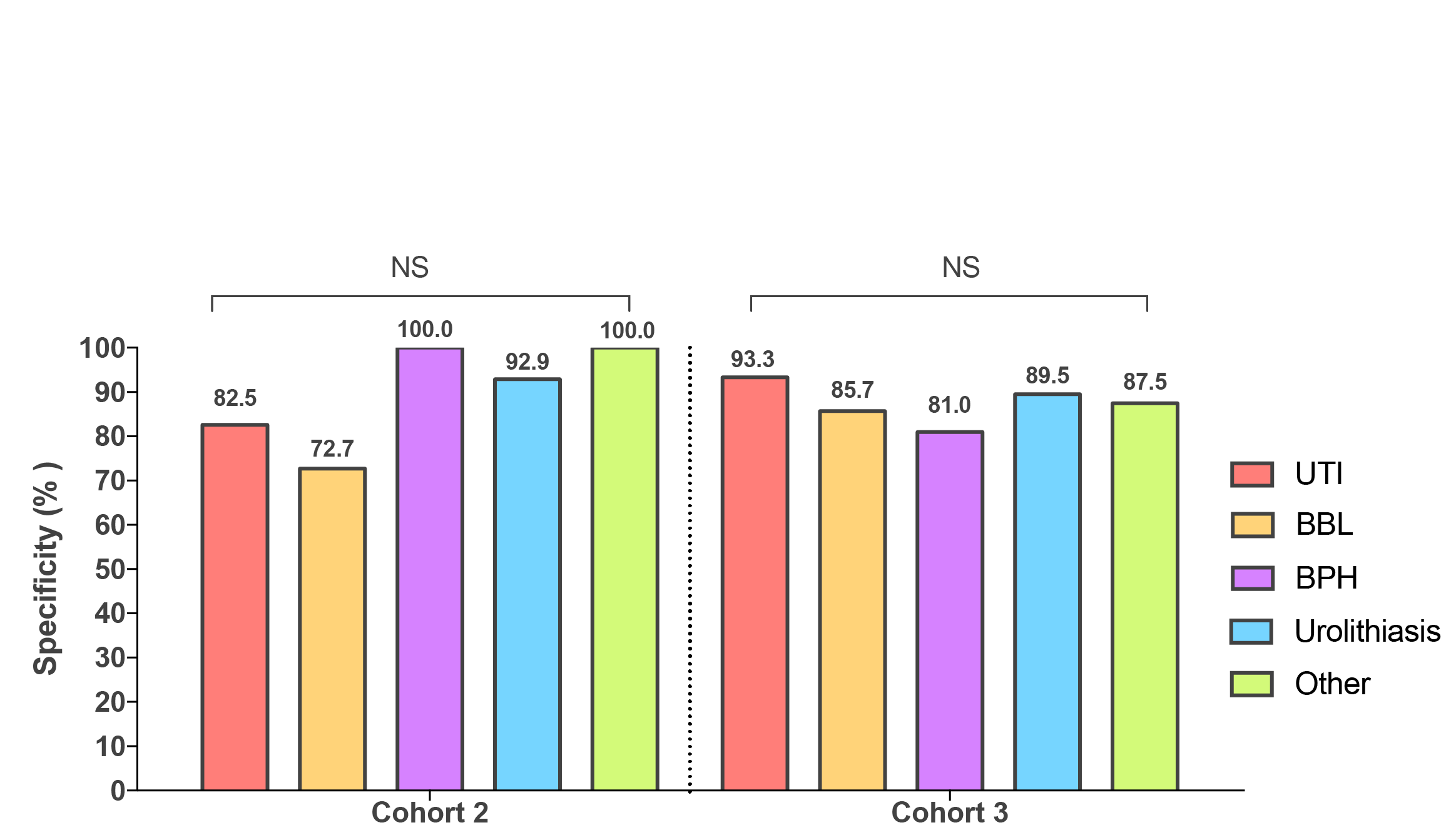

Supplement: Supplementary file 2 — Additional file 2: Figure S2. Specificities of the assay of dual-marker model in patients with different disorder conditions in cohorts 2 and 3. Statistical significance was assessed by χ2 test; NS, no statistical significance. [file 13148_2021_1073_MOESM2_ESM.tif]

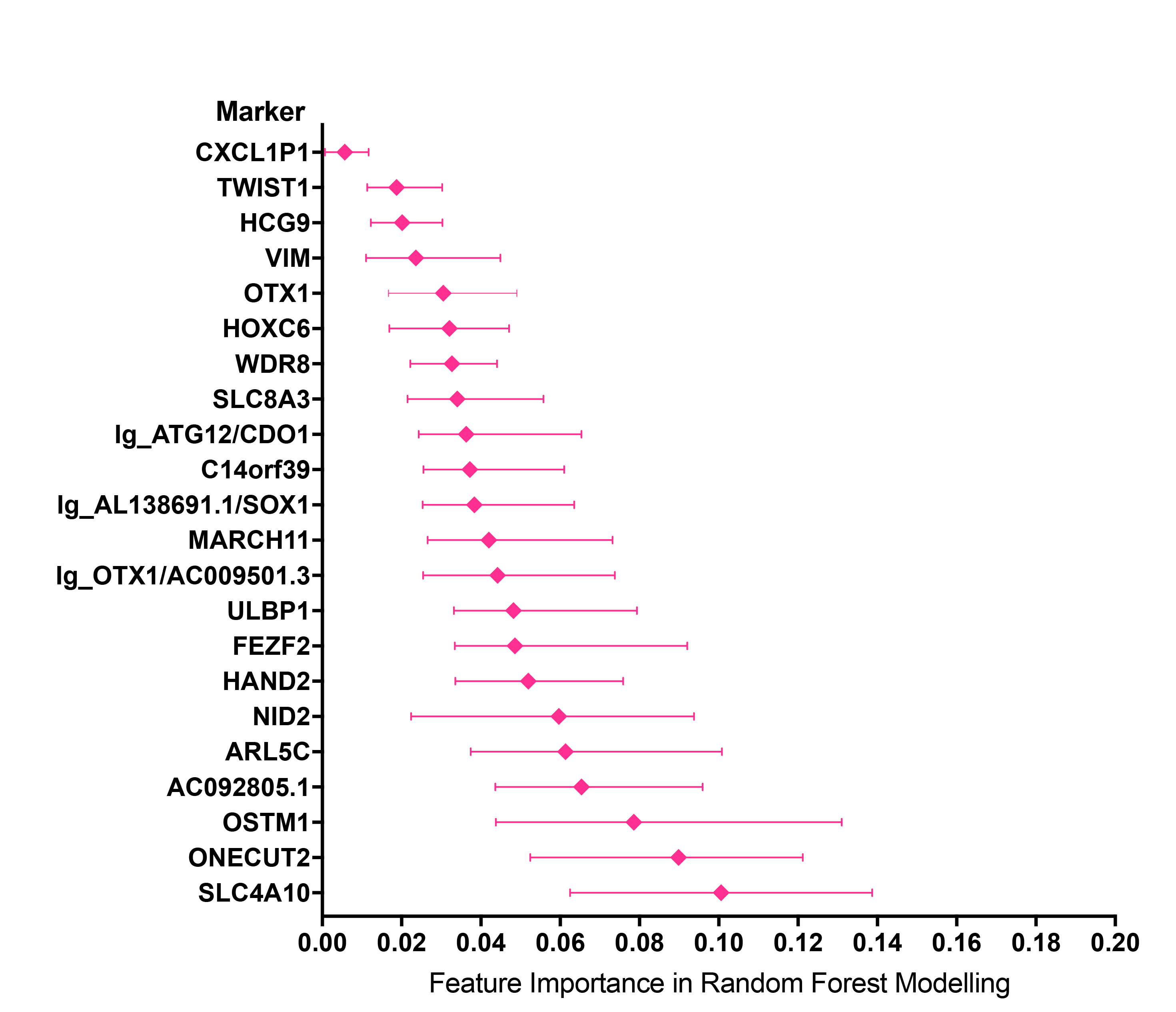

Supplement: Supplementary file 3 — Additional file 3: Figure S3. Feature importance of the 22 markers in random forest modeling in Cohort 1 for risk stratification of BC; random forest modeling was performed in 100 splits of train-test sampling in the cohorts and contributions of each markers on each of the models were scored as feature importance; the importance of each marker for classifying non-BC, LMR-NMIBC and HR-NMIBC+MIBC cases was expressed as mean with range in 100 splits of sampling. [file 13148_2021_1073_MOESM3_ESM.tif]

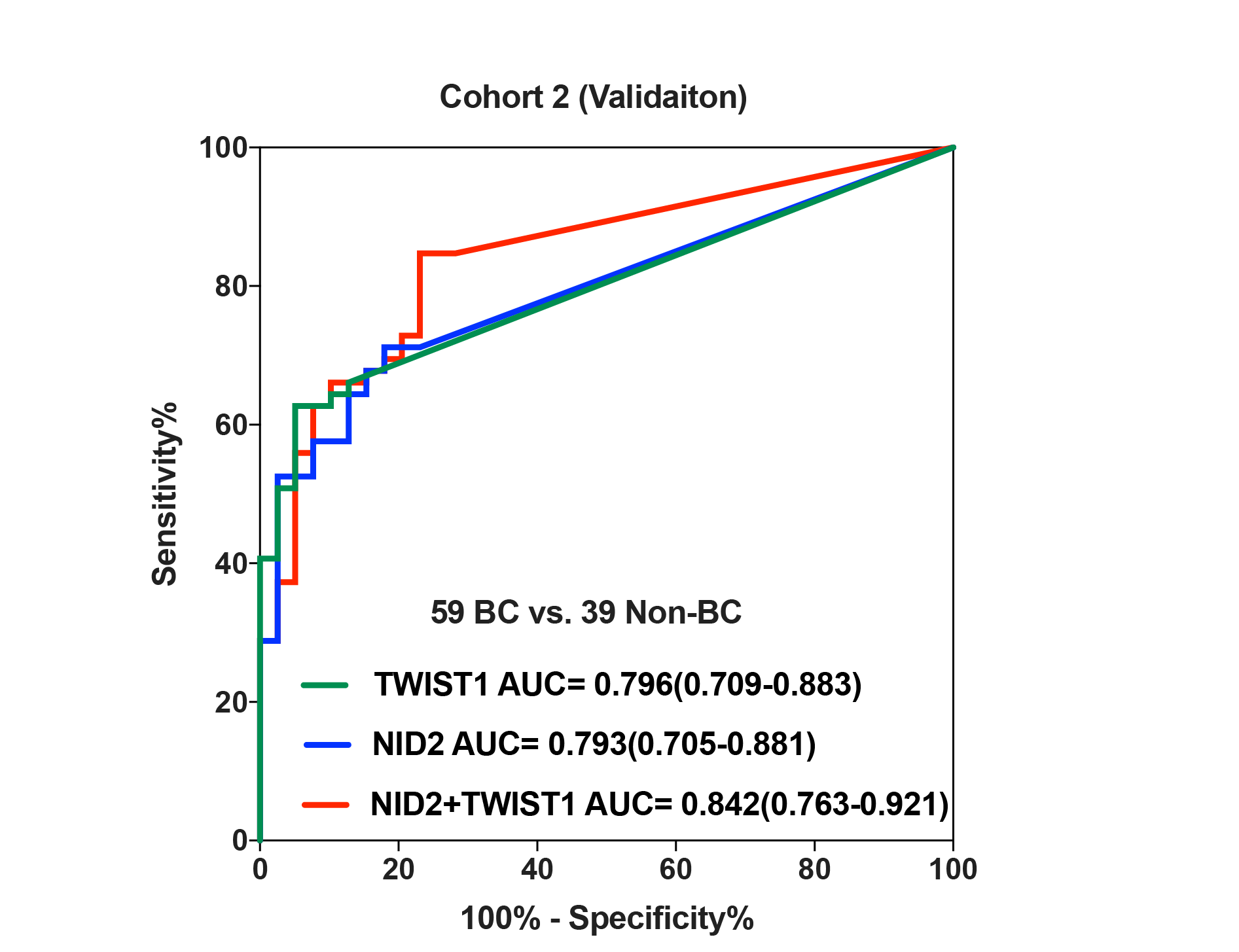

Supplement: Supplementary file 4 — Additional file 4: Figure S4. ROC curves of NID2, TWIST1 and model of combinations of NID2 and TWIST1 for detection of BC in cohort 2. BBL, bladder benign lesions; BPH, benign prostatic hyperplasia; UTI, urinary tract infections. [file 13148_2021_1073_MOESM4_ESM.tif]

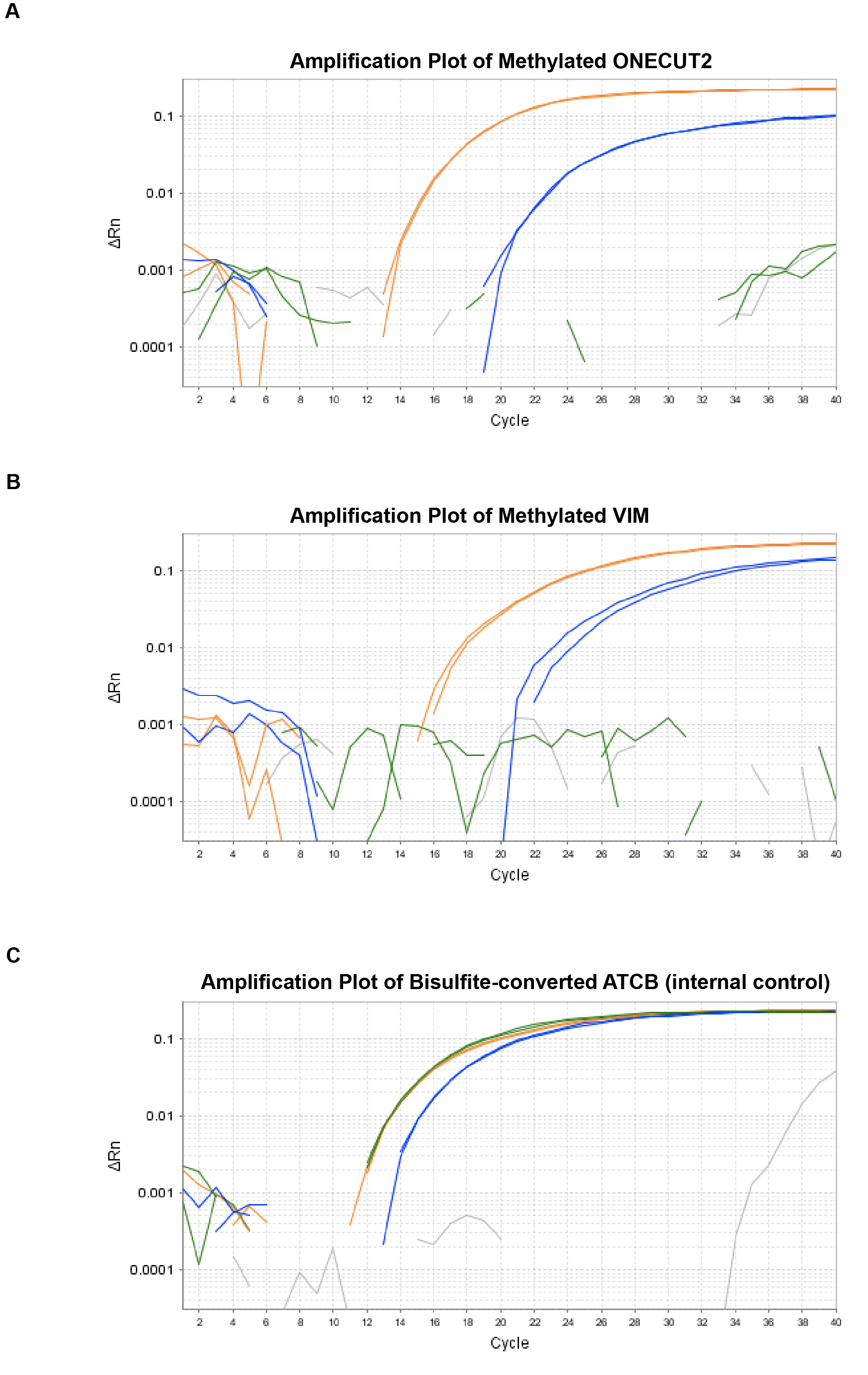

Supplement: Supplementary file 6 — Additional file 6: Figure S5. Amplification curves of selected markers and internal controls in the methylation assay. Amplification signals were expressed as ∆Rn, where Rn was the target fluorescent signal normalized to the signal of the passive reference dye and ∆Rn was the Rn value minus that of the instrument baseline signal. A, Amplification curves of a methylated bisulfite-converted DNA fragment of ONECUT2 in a randomly selected case (blue lines), positive control (orange lines), negative control (green lines) and NTC control (gray line); B, amplification curves of a methylated bisulfite-converted DNA fragment of VIM in a randomly selected case (blue lines), positive control (orange lines), negative control (green lines) and NTC control (gray line); C, amplification curves of a DNA fragment of ATCB as a control for the measurement of total bisulfite-treated methylated and unmethylated DNA molecules in a randomly selected case (blue lines), positive control (orange lines), negative control (green lines) and NTC control (gray line). [file 13148_2021_1073_MOESM6_ESM.tif]
