# Supplementary material for: A urine-based DNA methylation assay to facilitate early detection and risk stratification of bladder cancer
Source: Clin Epigenetics. 2021 Apr 26;13:91. doi: 10.1186/s13148-021-01073-x (PMC8072728; doi:10.1186/s13148-021-01073-x)
Supplement: Supplementary file 5 — Additional file 5. Supplementary information. [file 13148_2021_1073_MOESM5_ESM.docx]

**A urine-based DNA methylation assay to facilitate early detection and risk stratification of bladder cancer**

Weimei Ruan^1a^, Xu Chen^2a^, Ming Huang^2^, Hong Wang^1^, Jiaxin Chen^1^, Zhixin Liang^1^, Jingtong Zhang^2^, Yanqi Yu^3^, Shang Chen^3^, Shizhong Xu^2^, Tianliang Hu^1^, Xia Li^1^,Yuanjie Guo^1^, Zeyu Jiang^1^ , Zhiwei Chen^1,4*^, Jian Huang^2, 5*^, Tianxin Lin^2, 5*^, Jian-Bing Fan^3,1*^

^1^AnchorDx Medical Co., Ltd, Unit 502, 3^rd^ Luoxuan Road, International Bio-Island, Guangzhou, China 510300

^2^ Department of Urology, Sun Yat-sen Memorial Hospital, Sun Yat-sen University, Guangzhou, China 510120

^3^ Department of Pathology, School of Basic Medical Science, Southern Medical University, Guangzhou, China 510515

^4^ AnchorDx, Inc., 46305 Landing Pkwy, Fremont, CA, United States 94538

^5^ Guangdong Provincial Key Laboratory of Malignant Tumor Epigenetics and Gene Regulation, Sun Yat-sen Memorial Hospital, Sun Yat-sen University, Guangzhou, China 510120

**Supplementary Figure legends**

**Supplementary Figure 1**. Feature importance of the 22 markers in random forest modelling in Cohort 1 for BC detection; Random forest modelling was performed in 100 splits of train-test sampling in the cohorts and contributions of each markers on each of the models were scored as feature importance; The importance of each marker for classifying BC and Non-BC cases was expressed as mean with range in 100 splits of train-test sampling.

**Supplementary Figure 2**. Specificities of the assay of dual-marker model in patients with different disorder conditions in cohort 2 and 3. Statistical significance was assessed by χ2 test; NS, no statistical significance.

**Supplementary Figure 3**. Feature importance of the 22 markers in random forest modelling in Cohort 1 for risk stratification of BC; Random forest modelling was performed in 100 splits of train-test sampling in the cohorts and contributions of each markers on each of the models were scored as feature importance; The importance of each marker for classifying Non-BC, LMR-NMIBC and HR-NMIBC+MIBC cases was expressed as mean with range in 100 splits of sampling.

**Supplementary Figure 4**. ROC curves of NID2, TWIST1 and model of combinations of NID2 and TWIST1 for detection of BC in Cohort 2. BBL, bladder benign lesions; BPH, benign prostatic hyperplasia; UTI, urinary tract infections.

**Supplementary Figure 5**. Amplification curves of selected markers and internal controls in the methylation assay. Amplification signals were expressed as ∆Rn, where Rn was the target fluorescent signal normalized to the signal of the passive reference dye and ∆Rn was the Rn value minus that of the instrument baseline signal. **A**, Amplification curves of a methylated bisulfite-converted DNA fragment of ONECUT2 in a randomly selected case (blue lines), positive control (orange lines), negative control (green lines) and NTC control (grey line); **B**, Amplification curves of a methylated bisulfite-converted DNA fragment of VIM in a randomly selected case (blue lines), positive control (orange lines), negative control (green lines) and NTC control (grey line); **C**, Amplification curves of a DNA fragment of ATCB as a control for the measurement of total bisulfite-treated methylated and unmethylated DNA molecules in a randomly selected case (blue lines), positive control (orange lines), negative control (green lines) and NTC control (grey line).

**Supplementary Tables**

**Supplementary Table 1 Patient characteristics in the Cohort 1 and 2**

|  | Cohort 1 (model development) | | Cohort 2 (validation) | |
| --- | --- | --- | --- | --- |
| Characteristics | Groups | | | |
|  | BC | Non-BC | BC | Non-BC |
| Number of participants | 118 | 86 | 63 | 40 |
| Measurement available | 116 | 76 | 59 | 39 |
| Age [Mean (range), years] | 63 (17-83) | 58 (21-97) | 63 (39-83) | 56 (22-83) |
| Gender |  |  |  |  |
| Female (n, %) | 21 (18.1%) | 25 (32.9%) | 12 (20.3%) | 12 (30.8%) |
| Male (n, %) | 95 (81.9%) | 51 (67.1%) | 47 (79.7%) | 27 (69.2%) |
| T Staging |  |  |  |  |
| Tis (n, %) | - | - | 1(1.7%) | - |
| Ta (n, %) | 30(25.9%) | - | 18 (30.5%) | - |
| T1 (n, %) | 38(32.8%) | - | 16 (27.1%) | - |
| T2 (n, %) | 20 (17.2%) | - | 7 (11.9%) | - |
| T3 (n, %) | 22 (19.0%) | - | 13 (22.0%) | - |
| T4 (n, %) | 5 (4.3%) | - | 2 (3.4%) | - |
| Not available (n, %) | 1(0.9%) | - | 2(3.4%) | - |
| Tumor Grades |  |  |  |  |
| Low grade (n, %) | 21 (18.1%) | - | 9 (15.3%) | - |
| High grade (n, %) | 95 (81.9%) | - | 48 (81.4%) | - |
| Not available (n, %) | - | - | 2(3.4%) | - |
| Invasiveness |  |  |  |  |
| NMIBC (n, %) | 68 (58.6%) | - | 35 (59.3%) | - |
| MIBC (n, %) | 47 (40.5%) | - | 22 (37.2%) | - |
| Not available (n, %) | 1 (0.9%) | - | 2(3.4%) | - |
| NMIBC risk |  |  |  |  |
| Low-intermediate risk (n, %) | 26 (38.2%) | - | 14 (40.0%) | - |
| High risk (n, %) | 42 (61.8%) | - | 20 (57.1%) | - |
| Not available (n, %) | - | - | 1 (2.9%) | - |
| Urine FISH |  |  |  |  |
| Positive(n, %) | 77 (66.4%) | 2 (2.6%) | 38 (64.4%) | 1 (2.6%) |
| Negative(n, %) | 32 (27.6%) | 22 (28.9%) | 15 (25.4%) | 10 (25.6%) |
| Not available(n, %) | 7 (6.0%) | 52 (68.4%) | 6 (10.2%) | 28 (71.8%) |
| Urine cytology |  |  |  |  |
| Positive(n, %) | 58 (50.0%) | 2 (2.6%) | 31 (52.5%) | 1 (2.6%) |
| Negative(n, %) | 50 (43.1%) | 22 (28.9%) | 23 (39.0%) | 8 (20.5%) |
| Not available(n, %) | 8 (6.9%) | 52 (68.4%) | 5 (8.5%) | 30 (76.9%) |

FISH, fluorescence in situ hybridization; NMIBC, non-muscle invasive bladder cancer; MIBC, muscle invasive bladder cancer.

**Supplementary Table 2 Patient characteristics in the Cohort 3**

|  | Cohort 3 (in hematuria population) | |
| --- | --- | --- |
| Characteristics | BC | Non-BC |
| Number of participants | 35 | 147 |
| Sample meet study criteria | 34 | 140 |
| Age [Mean (range), years] | 65 (37-88) | 55 (20-85) |
| Gender |  |  |
| Female (n, %) | 7 (20.6%) | 47 (33.6%) |
| Male (n, %) | 27 (79.4%) | 93 (66.4%) |
| T Staging |  |  |
| Tis (n, %) | 2 (5.9%) | - |
| Ta (n, %) | 12 (35.3%) | - |
| T1 (n, %) | 16 (47.1%) | - |
| T2 (n, %) | 3 (8.8%) | - |
| T3 (n, %) | 1 (2.9%) | - |
| T4 (n, %) | 1 (2.9%) | - |
| Tumor Grades |  |  |
| Low grade (n, %) | 6 (17.6%) | - |
| High grade (n, %) | 28 (82.4%) | - |
| Invasiveness |  |  |
| NMIBC (n, %) | 29 (85.3%) | - |
| MIBC (n, %) | 5 (14.7%) | - |
| Urine FISH |  |  |
| Positive(n, %) | 19 (55.9%) | 6 (4.3%) |
| Negative(n, %) | 8 (23.5%) | 30 (21.4%) |
| Not available(n, %) | 7 (20.6%) | 104 (74.3%) |
| Urine cytology |  |  |
| Positive(n, %) | 14 (41.2%) | 5 (3.6%) |
| Negative(n, %) | 13 (38.2%) | 30 (21.4%) |
| Not available(n, %) | 7 (20.6%) | 105 (75.0%) |

FISH, fluorescence in situ hybridization; NMIBC, non-muscle invasive bladder cancer; MIBC, muscle invasive bladder cancer.

**Supplementary Table 3 Genomic information of the 22 methylation markers and internal control**

| Marker ID | Gene Name | Chromosome location (hg19) | Sequences of the Gene Regions  ([CG] denotes the involved methylation sites) |
| --- | --- | --- | --- |
| 01 | OTX1 | chr2:63283954-63284069 | CTTCAGCTGCCCT[CG]ATTTTGCTCCA[CG]CCTGC[CG]GCCAGAGCCTCC[CG]G[CG]TTTCTTC[CG]CCCCAG[CG]GAGTG[CG]CTGGGG[CG][CG]CCAGGGCTAGGCC[CG]C[CG]GAGGAG[CG][CG]TC |
| 02 | HAND2 | chr4:174450329-174450410 | C[CG]GTGGTGCACCA[CG]AGGGCTACC[CG]TTTGC[CG]C[CG]C[CG]C[CG]C[CG]CAGCTGC[CG]C[CG]C[CG]C[CG]CCAGC[CG]CTGCAGCCATG |
| 03 | Ig_AL138691.1/SOX1 | chr13:112710589-112710674 | CTCC[CG][CG]CCCC[CG]CCCC[CG][CG]TTC[CG]GCCTGGCCTG[CG]GGATT[CG]GGC[CG]AGGCAACTGCAGGGA[CG]GGGCACCCCTCCTGCTCC |
| 04 | VIM | chr10:17271461-17271548 | ATGTT[CG]G[CG]GCC[CG]GGCAC[CG][CG]AGC[CG]GC[CG]AGCTCCAGC[CG]GAGCTA[CG]TGACTA[CG]TCCACC[CG]CACCTACAGCCTGGGCAGC |
| 05 | MARCH11 | chr5:16180072-16180160 | TGGAGAGGGGTCATC[CG]CCC[CG]GAAC[CG]A[CG]TGAG[CG][CG]GGGC[CG]GCC[CG]TGGAGG[CG]GCTGAGGGATCCCCCACTTCCAGCC[CG]CC[CG] |
| 06 | FEZF2 | chr3:62356910-62356998 | TGC[CG]CAAAATT[CG]CAGA[CG]AAGGGCTTGTAGCC[CG][CG]TGGATG[CG]GATATG[CG]TGTTGAG[CG]TGGAGCTG[CG]GTTGAA[CG]CTTTGC[CG] |
| 07 | WDR8 | chr1:3567301-3567373 | [CG][CG]GCCAC[CG]CC[CG]TTCATCACC[CG][CG][CG]CATCTGGGCTGGCAC[CG]GG[CG]AAGAAT[CG]TG[CG]GGTCTGGGAC |
| 08 | OSTM1 | chr6:108440261-108440368 | TTGG[CG]GTCAAAGTGGCCC[CG]ACT[CG]GGATGACAATTGA[CG]GGGATCAAGGGATTGCCCATTCTGTGCCTGTAAGAAC[CG]ATT[CG]TGCCAGAGAAACTCATCAAGTGG |
| 09 | HCG9 | chr6:29943403-29943495 | GCAAC[CG]GCAG[CG]TCCAGCTCC[CG]CACCT[CG]CTGCACAT[CG]CACCTGAGCCC[CG]C[CG][CG]AC[CG]CAT[CG][CG]CT[CG]CTG[CG]ACCCATTCAGACCC |
| 10 | CXCL1P1 | chr4:74809900-74810008 | TGCTACCCCAGC[CG]TGTCC[CG]CTC[CG]GAGACCCCAGGG[CG]C[CG]GGACCCATCTGC[CG]CT[CG]C[CG]GC[CG]GAGGCTACCAGGAGCAGGAGCAGCAG[CG]C[CG]CC[CG]CAGTAG |
| 11 | [C14orf39](https://useast.ensembl.org/Homo_sapiens/Gene/Summary?db=core;g=ENSG00000179008;tl=uF9kRG60Y5a8I3NH-6157411-770496259) | chr14:60973433-60973533 | [CG]TGTCTGAGGCT[CG][CG]GGCAACTGGAACTGAGAGTCTGAGTTGGCCT[CG][CG]GGAGC[CG]CCAGAAGGGTG[CG]GGCTG[CG]TGTGGCAGAGTAGGAGCACTGT |
| 12 | SLC4A10 | chr2:162283698-162283786 | [CG]C[CG]GGCTCCAGGGCTCC[CG][CG]CTCCAGTGGCCCAGCCTGGG[CG]GAGAGCAGAG[CG][CG]GCCCC[CG][CG]GCCC[CG][CG]GCCT[CG]AGCCC[CG] |
| 13 | ARL5C | chr17:37321479-37321562 | CC[CG]GAGTGGGGCAGGTGT[CG]GAGCTGGGTGGGAAGCAGA[CG][CG]GTA[CG]GTGGGCAGAGGTCCCCAGCCTG[CG]GGGAG[CG]CTAT |
| 14 | HOXC6 | chr12:54408647-54408739 | GGAGAGAAAGTCCTATCTGCAGCAGC[CG]AATGGTCCCCATTC[CG]GTAATGGGA[CG]G[CG]GGAGCATTTGGGAGGA[CG][CG]ATTCTAAAGAGAG[CG] |
| 15 | ULBP1 | chr6:150286446-150286532 | GCAGCAGCTGCAGGAAG[CG]GACT[CG]G[CG]GAAAGGAGCCC[CG]GAGGGGAACTGAGTGCCTTCAGCCAGGCA[CG]TT[CG]GGGAGACAG[CG] |
| 16 | Ig_OTX1/AC009501.3 | chr2:63285431-63285529 | AAAACTGATC[CG]TGTCCTGCATGTTGGCAGCAGACAACCTTCCTTGCTGCTGAGCTGTCC[CG]GGTGGCTTCAC[CG][CG]GCTGGGGAATC[CG]AGCCATTCC |
| 17 | SLC8A3 | chr14:70653656-70653766 | [CG]GCCTTC[CG]GTGGGGCACCAAAAGGGAAGCCTCCT[CG]GCCCCTGG[CG]ACC[CG]GTGACTTGCAG[CG]G[CG]TGTGATTAATCTTCCACAGCTGT[CG]TGCCCCATCCACTTGAG |
| 18 | AC092805.1 | chr1:91185422-91185527 | [CG]GGAACTGAGTGCTGGCC[CG]GGAGACCCTC[CG]GAGAGCT[CG][CG]GGCT[CG]GCCT[CG]GCCT[CG]GCCT[CG]GCCTT[CG]GC[CG][CG]GTTAC[CG]AAACACAGA[CG]GTAGACT |
| 19 | NID2 | chr14:52535902-52535986 | T[CG]GCAGTGGCCACCACATCTGGTTCT[CG]TTAACTTTTCTAAGGCAG[CG]GC[CG]CTGGAGCAG[CG]GGGCTGG[CG]GGGTAAAAGCTC |
| 20 | ONECUT2 | chr18:55108771-55108863 | TAGGAAGACT[CG]GGCAC[CG]TTCAG[CG]CATTGGCTT[CG][CG]GACCCAGC[CG]CCCAGG[CG]GAT[CG]C[CG]GAAG[CG]CAAGTAG[CG]GTGTGTG[CG]CACAG |
| 21 | TWIST1 | chr7:19157564-19157656 | GGAG[CG]TG[CG]GGCAG[CG]CCCC[CG]AACCCTAG[CG]CAGCCCAGGAAG[CG]GT[CG]GAGGAGACTGTCCTGGC[CG][CG]GTGGCAGCCCCATC[CG]GAGTG |
| 22 | Ig_ATG12/CDO1 | chr5:115152472-115152583 | AGATTG[CG][CG]GAGCCCA[CG][CG]ATCCCTGGGA[CG]C[CG]GAGACAA[CG]GGGCTCTTGGGAAGG[CG][CG]GAGCC[CG]GGGAAGC[CG]GGGATGTG[CG][CG]TGAGC[CG]TGCC[CG]CAGGGTC |
| Internal control | ATCB | chr7: 5571731- 5571859 | CCAATAAAACCTACTCCTCCCTTAAAAATTACAAAAACCACAACCTAATAAAAAAAATAACCACCACCCAACACACAATAACAAACACAAATTCACAATCCAAAAAACTTACTAAACCTCCTCCATCAC |

An intergenic region is annotated as Ig_gene1/gene2, where gene 1 and gene 2 are the two adjacent genes of the intergenic region.
